# Effective Capture of Carbon Dioxide Using Hydrated Sodium Carbonate Powders

**DOI:** 10.3390/ma11020183

**Published:** 2018-01-24

**Authors:** Yuanhao Cai, Weilin Wang, Liang Li, Zhaofeng Wang, Suying Wang, Hao Ding, Zhengguo Zhang, Luyi Sun, Weixing Wang

**Affiliations:** 1 School of Chemistry and Chemical Engineering, China Key Laboratory of Enhanced Heat Transfer and Energy Conservation of the Ministry of Education, South China University of Technology, Guangzhou 510640, Guangdong, China; ceyhcai@163.com (Y.C.); weilinwang92@gmail.com (W.W.); liliang628.scut@gmail.com (L.L.); cewangsuying@mail.scut.edu.cn (S.W.); cezhang@scut.edu.cn (Z.Z.); 2 Polymer Program, Institute of Materials Science, University of Connecticut, Storrs, CT 06269, USA; zhfwang@licp.cas.cn (Z.W.); hao.2.ding@uconn.edu (H.D.); 3 Department of Chemical and Biomolecular Engineering, University of Connecticut, Storrs, CT 06269, USA; 4 Department of Biomedical Engineering, University of Connecticut, Storrs, CT 06269, USA

**Keywords:** hydrated sodium carbonate, CO_2_ capture, free-flowing powders

## Abstract

The emission of CO_2_ has been considered a major cause of greenhouse effects and global warming. The current CO_2_ capture approaches have their own advantages and weaknesses. We found that free-flowing hydrated sodium carbonate (Na_2_CO_3_) powders with 30 wt % water can achieve a very high CO_2_ sorption capacity of 282 mg/g within 60 min and fast CO_2_ uptake (90% saturation uptake within 16 min). The results suggest that the alkaline solution resulting from the dissolution of partial Na_2_CO_3_ can freely attach onto the hydrated Na_2_CO_3_ particles, which provides an excellent gas–liquid interface for CO_2_ capture, leading to significantly enhanced CO_2_ sorption capacity and kinetics.

## 1. Introduction

Emission of CO_2_ is identified as the main contributor to global climate change. Reducing the levels of CO_2_ in the atmosphere has become a pressing issue worldwide, and capturing and sequestrating CO_2_ as an option to decrease levels of CO_2_ has been widely explored [1,2,3,4].

A number of promising materials for CO_2_ capture were reported [5,6,7,8,9,10]. The best developed are probably aqueous amines [11,12], including monoethanolamine (MEA) [13,14] and diethanolamine (DEA) [15,16]. However, liquid amines have some serious disadvantages, including amine evaporation [17,18], corrosion to equipment [19], and high energy cost for regeneration [20,21]. A feasible way to reduce the corrosivity and the regeneration energy is to use supported amine adsorbents [22,23,24,25,26], but the raw materials are currently too expensive to be applied in large-scale industrial settings [27].

As an alternative to supported amine sorbents, alkali metal carbonates such as K_2_CO_3_ and Na_2_CO_3_ as solid sorbents have received wide attention with both high sorption capacity and low cost [28,29,30,31,32]. However, the main problem of using carbonates is their slow reaction kinetics [33,34,35]. Cooper and co-workers reported that dry K_2_CO_3_ solution (K_2_CO_3_ aqueous solution coated with hydrophobic silica powders) exhibited significantly increased CO_2_ uptakes [36], but the recyclability of this sorbent was poor. It has been generally accepted that K_2_CO_3_ is superior to Na_2_CO_3_ in terms of both CO_2_ uptake capacity and kinetics [37,38,39]. However, using Na_2_CO_3_ will be more competitive for large-scale industrial applications because of its lower cost, especially if one can dramatically promote the rate of the key reaction:

Na_2_CO_3_ + H_2_O + CO_2_ ⇌ 2NaHCO_3_ (1)

One of the most common approaches to tackle this problem is to disperse Na_2_CO_3_ powders on solid supports [40,41], but such a strategy also reduces CO_2_ sorption capacity because the inclusion of the supports greatly decreases the amount of active components per unit mass [42].

In this report, we demonstrate that support-free hydrated sodium carbonate powders (HSCPs) prepared by simply mixing a certain amount of water and Na_2_CO_3_ powders exhibit effective CO_2_ capture. The alkaline solution resulting from the dissolution of partial Na_2_CO_3_ can freely attach into hydrated Na_2_CO_3_ particles, which provides an excellent gas–liquid interface for CO_2_ capture, leading to significantly enhanced CO_2_ sorption capacity and kinetics. The elimination of supports not only reduces the overall cost of raw materials, but also increases the CO_2_ sorption capacity, both of which are critical for large-scale applications.

## 2. Experimental

### 2.1. Preparation of HSCPs

Na_2_CO_3_ (99.8%) was purchased from Tianjin Qilun Chemical Technology Co. Ltd., Tianjin, China. Na_2_CO_3_·H_2_O (99%) was purchased from Aladdin Co. Ltd., Shanghai, China. MEA (99%) was purchased from Jiangsu Yonghua Chemical Technology Co. Ltd., Changshu, China. CO_2_ (99.9%) was supplied by Zhuozheng Gas Co. Ltd., Guangzhou, China. All the chemicals were used as received without further purification. A series of HSCPs with different Na_2_CO_3_ contents were prepared by thoroughly mixing an appropriate amount of Na_2_CO_3_ and deionized water at room temperature.

### 2.2. Characterization

X-ray diffraction (XRD) patterns of the samples were recorded using a Bruker D8 diffractometer (Bruker, Karlsruhe, Germany) with Bragg–Brentano θ−2θ geometry (20 kV and 5 mA), using a graphite monochromator with Cu Kα radiation.

To measure the CO_2_ capture capacity of the HSCP samples, 5.0 g HSCP was charged into a 50 mL container, which was exposed to CO_2_ using a balloon containing a sufficient amount of CO_2_ gas (ca. 5 L with a pressure of ca. 1.05 bar). The amount of CO_2_ captured by each HSCP sample was measured using a balance. A muffle furnace (Luoyang BSK Electronic Materials Co. Ltd., Luoyang, China) was used to regenerate the sorbents at 250 °C for 1 h, which was mixed with water to reform HSCPs.

## 3. Results and Discussion

**Figure 1:**
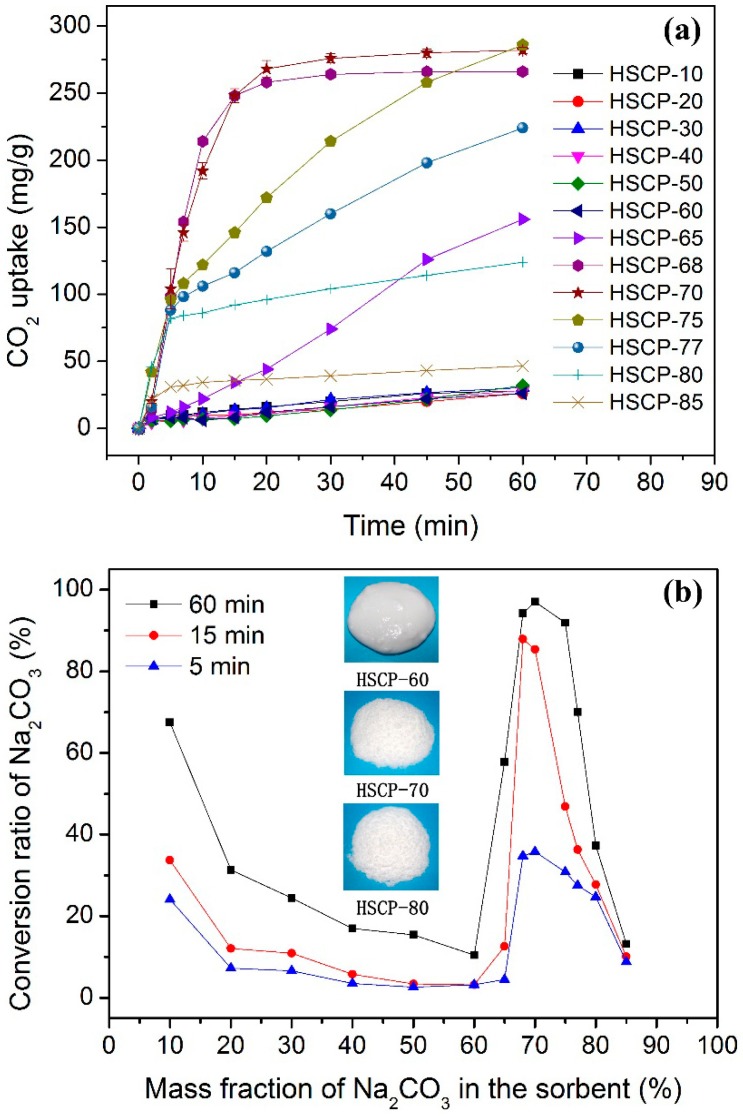
(**a**) CO_2_ sorption kinetics of various HSCPs at 30 °C; (**b**) conversion ratio of Na_2_CO_3_ in various HSCPs and different reaction time.

Figure 1a shows the CO_2_ uptake kinetic curves using various HSCPs (labelled as HSCP-X, where X is the mass percentage of Na_2_CO_3_ in the mixture) as a sorbent at 30 °C. It was found that HSCP-10 to HSCP-60 had a very low CO_2_ sorption capacity (<32 mg/g of HSCP). The CO_2_ uptake capacity rapidly rose to 156 mg/g when the mass fraction of Na_2_CO_3_ was increased to 65 wt %, i.e., HSCP-65, but it still suffered from low sorption kinetics. Further increasing mass fraction of Na_2_CO_3_ led to another significant increase in term of both sorption capacity and kinetics. At the optimum concentration of 70 wt % (i.e., HSCP-70), the CO_2_ uptake capacity reached 282 mg/g within 60 min, and the t_90_ (the time to achieve 90% of this capacity) was only 16 min. This capacity is much higher than that of other Na_2_CO_3_-based CO_2_ sorbents reported in the literature, which varies between 32 and 140 mg/g [43,44]. Although HSCP-75 achieved the highest capacity (286 mg/g), its CO_2_ sorption rate was relatively slow and t_90_ was about 45 min.

It was found that too high a concentration of Na_2_CO_3_ in HSCP would actually lower the CO_2_ uptake capacity. When the concentration of Na_2_CO_3_ in HSCP reached 80 and 85 wt %, the CO_2_ uptake capacity decreased to 124 and 46 mg/g, respectively. Theoretically, the CO_2_ sorption capacity is directly related to the amount of Na_2_CO_3_ in HSCPs when the content of water is more than 14.5 wt % according to Equation (1). Thus, the conversion ratio of Na_2_CO_3_ is a good indicator of the CO_2_ sorption behaviour. As shown in Figure 1b, with an increasing mass fraction of Na_2_CO_3_, the conversion ratio of Na_2_CO_3_ decreased initially, then dramatically increased to a maximum value close to 100% before declining again. After 60 min of reaction, HSCP-70 exhibited the highest conversion rate (97.1%), which suggested that most of Na_2_CO_3_ was consumed. The HSCPs with a low mass fraction of Na_2_CO_3_, such as HSCP-10, also showed a high conversion ratio, in which Na_2_CO_3_ dissolved in water to form a solution, due to its high degree of hydrolysis [45]. However, their corresponding CO_2_ uptake capacity is low because of the limited amount of Na_2_CO_3_ presented (Figure 1a). The other two sets of data in Figure 1b represent the conversion ratios of Na_2_CO_3_ after five and 15 min of reaction. For HSCP-70, its conversion ratio increased rapidly from 5 to 15 min, but changed little from 15 to 60 min, which suggested that most of Na_2_CO_3_ was consumed within 15 min, thus showing a high reaction rate.

**Figure 2:**
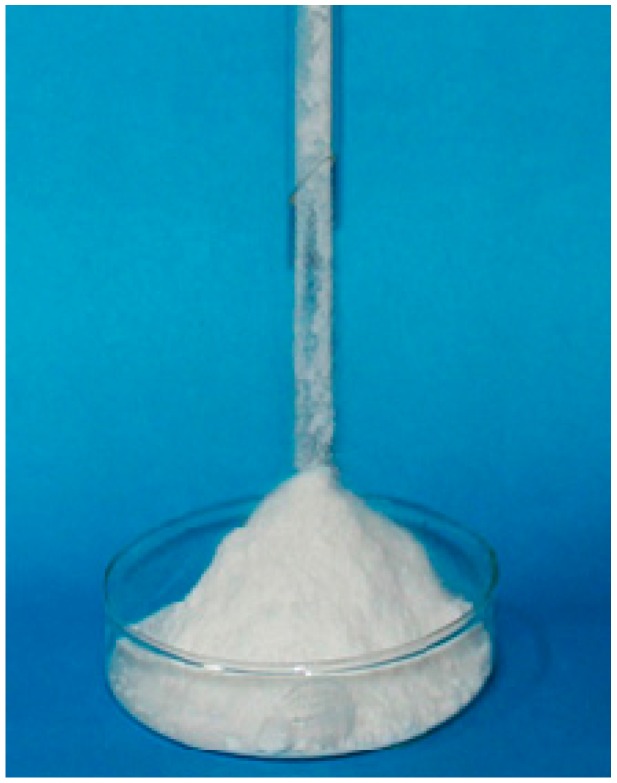
Free-flowing HSCP-70 from a glass funnel.

Overall, the above results show that the concentration of Na_2_CO_3_ in HSCPs has a great influence on CO_2_ capture, which can be explained by the fact that the morphology of HSCPs varies from aqueous solution and slurry, to powders with an increasing Na_2_CO_3_ concentration. At low Na_2_CO_3_ concentrations, the HSCPs exist as an aqueous solution or slurry as shown in Figure 1b (inset), which is not ideal for CO_2_ capture because of the low gas–liquid contact surface area. However, HSCP-70 is a sample of free-flowing powders (Figure 2) with a much higher gas–liquid contact surface area. This is why it has a rapid reaction rate and a high CO_2_ uptake capacity.

**Figure 3:**
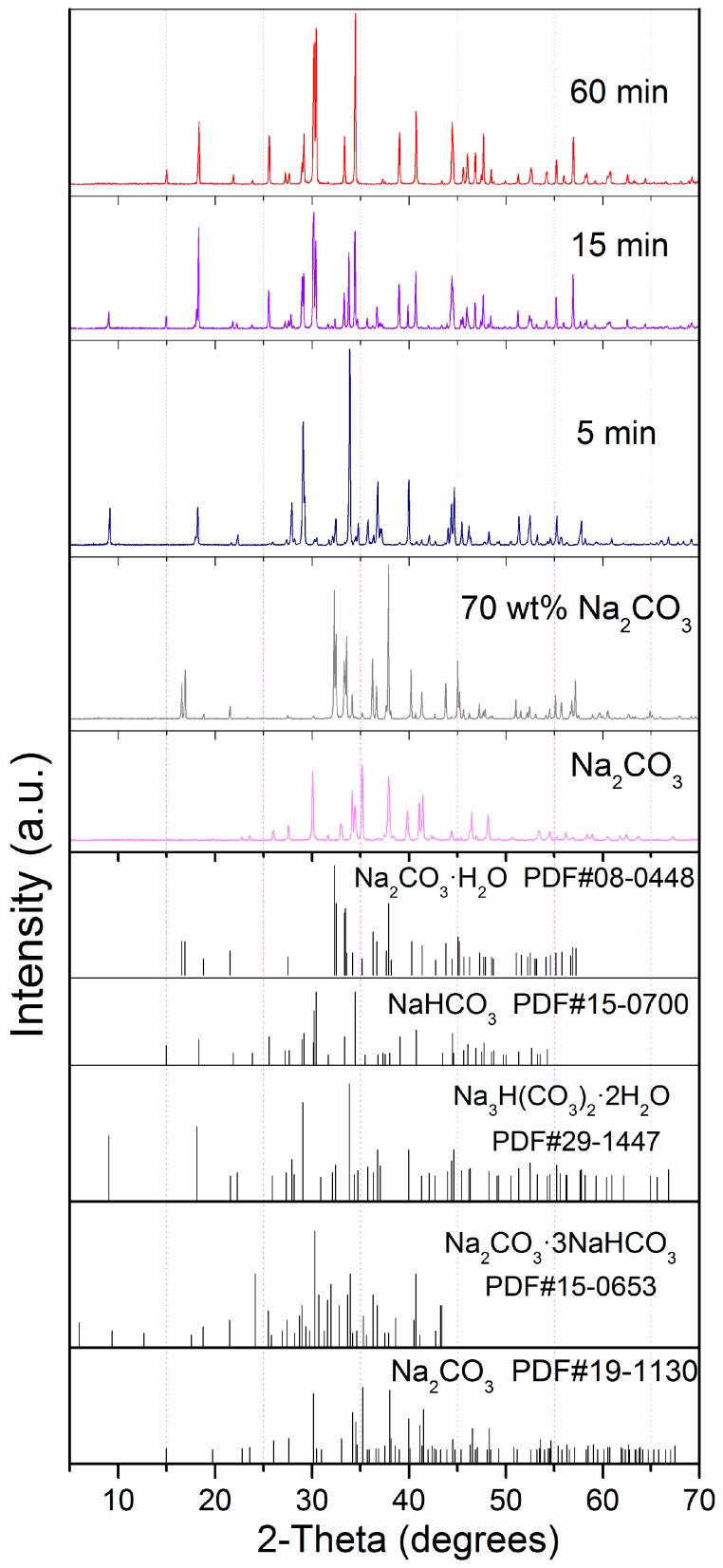
XRD patterns of various Na_2_CO_3_ based compounds and the reaction products of HSCP-70 after 0, 5, 15, and 60 min of CO_2_ sorption reaction at 30 °C.

**Figure 4:**
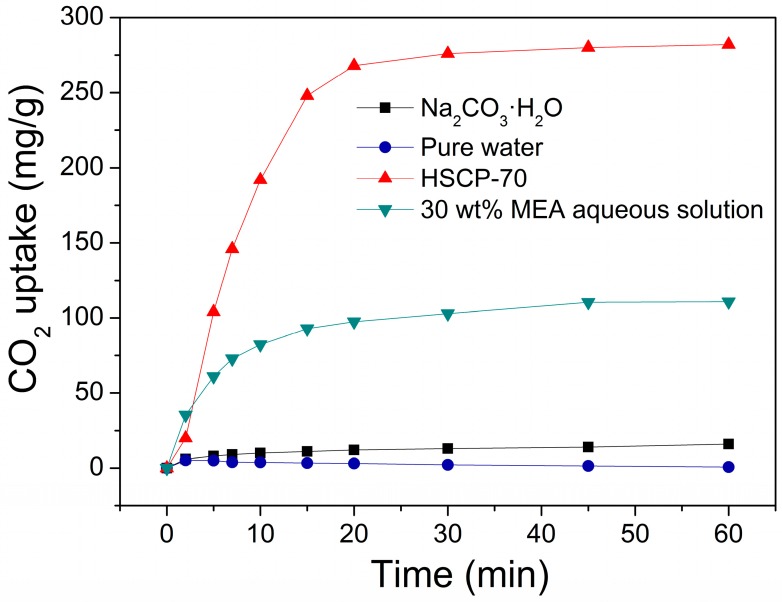
CO_2_ sorption kinetics of HSCP-70, pure water, 30 wt % MEA aqueous solution, and Na_2_CO_3_·H_2_O at 30 °C.

In order to better understand the mechanism of CO_2_ sorption by HSCPs, the XRD patterns (Figure 3) of various Na_2_CO_3_-based compounds were collected, including the reaction products of HSCP-70 after 0, 5, 15, and 60 min of sorption reaction at 30 °C. The XRD pattern of HSCP-70 was very close to the standard pattern of Na_2_CO_3_·H_2_O, which contains only 14.5 wt % water. This indicates that HSCP-70 contains extra water. As such, we also studied the CO_2_ sorption by pure Na_2_CO_3_·H_2_O, but it exhibited a low CO_2_ sorption capacity and rate (Figure 4). This suggests that the extra water contained in the sorbent plays a significant role in CO_2_ sorption. It indicates that the reaction proceeds most rapidly and effectively when Na_2_CO_3_, H_2_O, and CO_2_ are present simultaneously. Based on the above results, we propose that the extra water on the surface of HSCPs helps to form a basic alkaline aqueous environment. When CO_2_ diffuses to the surface of HSCPs, it reacts with the basic aqueous media. Since the reaction is exothermic, the generated heat triggers the decomposition of sodium carbonate hydrates, meanwhile releasing water to drive the reaction to proceed continuously. In addition, along the reaction of HSCP-70 and CO_2_, we also found that the characteristic peaks of Na_2_CO_3_ disappeared gradually, then intermediate structures, such as Na_3_H(CO_3_)_2_·2H_2_O (i.e., Na_2_CO_3_·NaHCO_3_·2H_2_O) and Na_2_CO_3_·3NaHCO_3_, appeared after reacting for five and 15 min, respectively. Eventually, virtually pure NaHCO_3_ formed after 60 min of reaction, which is expected.

The amine-based CO_2_ capture system is a proven technology that is already commercialized. To prevent excessive corrosion, typically 30 wt % MEA aqueous solution is used [11]. As shown in Figure 4, a 30 wt % MEA aqueous solution showed similar CO_2_ uptake kinetics initially, but its overall sorption capacity was relatively low (111 mg/g versus 282 mg/g for HSCP-70). We also studied the CO_2_ sorption capacity of pure water as a control, whose CO_2_ uptake capacity was ca. 0.7 mg/g (Figure 4).

**Figure 5:**
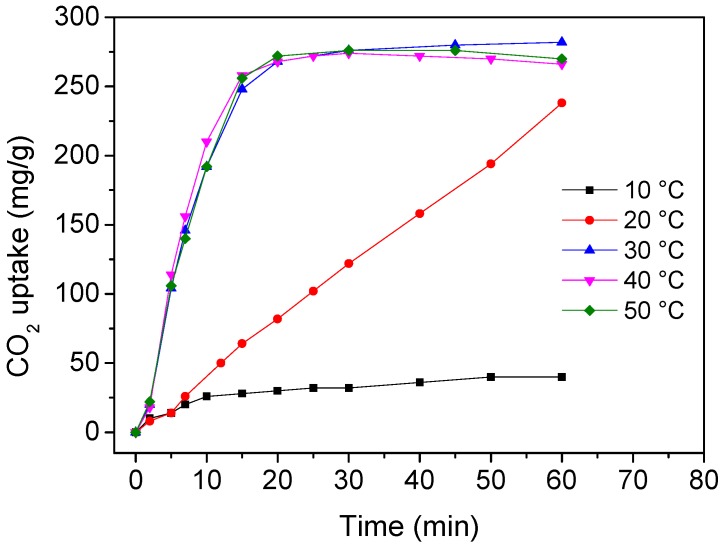
CO_2_ sorption kinetics of HSCP-70 at different temperatures.

**Figure 6:**
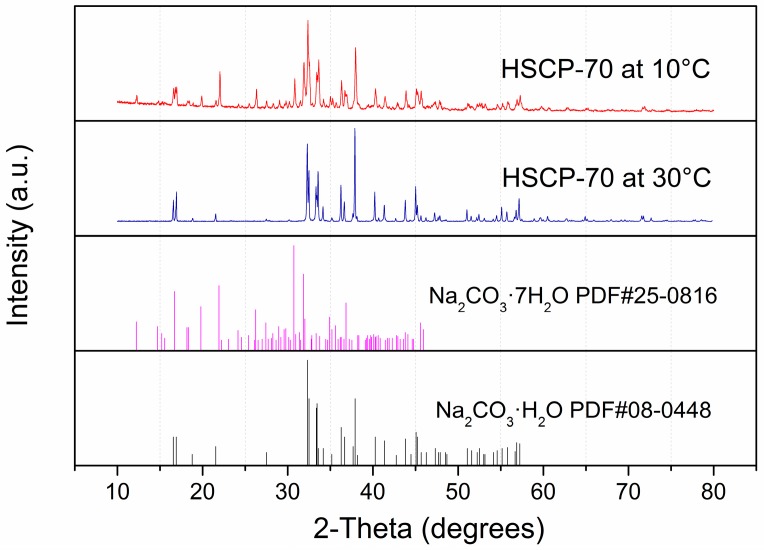
XRD patterns of HSCP-70 at 10 and 30 °C.

**Figure 7:**
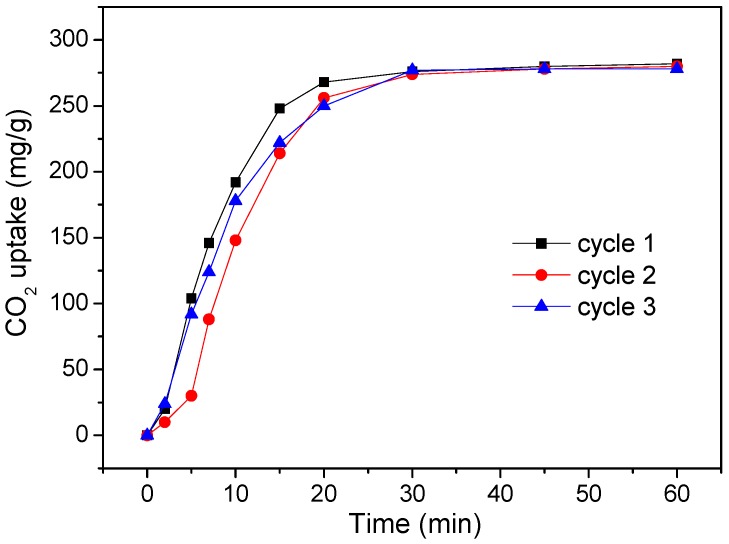
Recycling performance of HSCP-70 after regeneration at 250 °C.

We also studied the CO_2_ uptake kinetics at different temperatures and the recyclability of HSCP-70. The suitable temperature range for CO_2_ capture was determined to be 30–50 °C (Figure 5). A higher temperature will cause excessive evaporation of water in HSCP-70, and a lower temperature will cause the formation of Na_2_CO_3_·7H_2_O (as shown in Figure 6), both of which lead to a lower CO_2_ uptake of HSCP-70. HSCP-70 also exhibited excellent recyclability with little deterioration in CO_2_ sorption capacity and reaction rate after recycling (Figure 7).

## 4. Conclusions

In summary, we have demonstrated that support-free HSCPs can be used as effective sorbents for CO_2_ capture with a high capacity (282 mg/g) and fast sorption rate (90% saturation uptake within 16 min). The elimination of support and the low cost of Na_2_CO_3_ make this technology more competitive for large-scale applications. In addition, based on the reaction principles, HSCPs should also have high potential in capturing other acid gases, including SOx, NOx, H_2_S, and Cl_2_.
